# Cilostazol Suppresses Aβ-induced Neurotoxicity in SH-SY5Y Cells through Inhibition of Oxidative Stress and MAPK Signaling Pathway

**DOI:** 10.3389/fnagi.2017.00337

**Published:** 2017-10-17

**Authors:** Tatsunori Oguchi, Ran Ono, Mayumi Tsuji, Hidenobu Shozawa, Masayuki Somei, Manami Inagaki, Yukiko Mori, Taro Yasumoto, Kenjiro Ono, Yuji Kiuchi

**Affiliations:** ^1^Department of Pharmacology, School of Medicine, Showa University, Tokyo, Japan; ^2^Department of Internal Medicine, Division of Neurology, School of Medicine, Showa University, Tokyo, Japan; ^3^Department of Anesthesiology, Showa University Koto Toyosu Hospital, Tokyo, Japan

**Keywords:** amyloid β, cilostazol, Alzheimer’s disease, oxidative stress, neurotoxicity, apoptosis, MAPK, NADPH oxidase

## Abstract

Alzheimer’s disease (AD) is a slowly progressive form of dementia, characterized by memory impairment and cognitive dysfunction. AD is mainly characterized by the deposition of amyloid β (Aβ) plaques and intracellular neurofibrillary tangles in the brain, along with neuronal degeneration and high levels of oxidative stress. Cilostazol (CSZ) was recently found to suppress the progression of cognitive decline in patients with stable AD receiving acetylcholinesterase inhibitors. This present study aimed to clarify the mechanism by which CSZ protects neurons from degeneration associated with Aβ(1–42). We used Aβ(1–42) to induce neurotoxicity in human neuroblastoma SH-SY5Y cells. Cells were pretreated with CSZ before co-treatment with Aβ. To evaluate the effect of CSZ on oxidative stress, we examined levels of reactive oxygen species (ROS), nicotinamide adenine dinucleotide phosphate oxidase (Nox) activity, mRNA expression of NOX4, and Cu/Zn-Superoxide Dismutase (SOD), as well as apoptosis biomarkers [MTT, (3-(4,5-dimethylthiazol-2-yl)-2,5-diphenyltetrazolium bromide), caspase-3 and -9 activities and staining of annexin V]. We also assayed the activity of mitogen-activated protein kinases (MAPK): p38 MAPK and extracellular signal-regulated kinase1/2 (ERK1/2), and biomarkers of mitochondrial function (Bcl-2 and Bax), and cyclic adenosine monophosphate response element-binding protein (CREB). Aβ-induced oxidative stress (ROS, NOX4 activity, and expression of NOX mRNA), caspase activation (caspase-3 and -9), and p38 MAPK phosphorylation were suppressed by co-treatment with CSZ, but not by ERK1/2 activation. In addition, pretreatment with CSZ suppressed Aβ-induced apoptosis and increased cell viability via suppression of Bax (a proapoptotic protein), upregulation of Bcl-2 (an antiapoptotic protein) and Cu/Zn-SOD (a superoxide scavenging enzyme), and phosphorylation of CREB. These findings suggested that CSZ could counteract neurotoxicity through multiple mechanisms, one mechanism involving the attenuation of oxidative stress by suppressing NOX activity and Nox mRNA expression in Aβ-induced neurotoxicity and another involving the anti-neurotoxic effect via the ERK1/2/phosphorylated CREB pathway.

## Introduction

Alzheimer’s disease (AD) is the most frequently observed age-related neurodegenerative disease, which is clinically characterized by a progressive loss of memory in patients. The neuropathological features of AD include presence of amyloid plaques and neurofibrillary tangles, and loss of neurons and synapses in the brain regions associated with learning and memory (Kumar and Singh, 2015). According to the amyloid cascade hypothesis, amyloid β (Aβ) accumulation plays a central role in AD pathogenesis. Therefore, it is essential to elucidate the detailed mechanism of neuronal death induced by Aβ. Recent data indicate that apoptosis plays a critical role in neuronal degeneration of rats with AD (Calissano et al., 2009). Stimulation of neuronal apoptosis in AD brains promotes oxidative stress, which reduces mitochondrial redox activity (Radi et al., 2014). Furthermore, several studies suggest that Aβ-induced oxidative stress leads to neuronal apoptosis, which can be inhibited by antioxidants (Ding et al., 2015). These findings suggest that oxidative stress may be an important mediator of Aβ-induced neuronal cell death in the development of AD (Ferihan, 2013). However, the detailed mechanisms by which oxidative stress is associated with Aβ-induced neuronal degeneration in AD brains remain to be understood.

Mitogen-activated protein kinases (MAPKs) are a family of serine-threonine kinases that are activated by several factors and play a role in the regulation of intracellular signaling essential for cell proliferation, differentiation, survival, development, death and transformation (Dhillon et al., 2007). MAPK signaling pathways are related to the pathogenesis of a variety of neurodegenerative diseases such as AD, Parkinson’s disease and amyotrophic lateral sclerosis (Kim and Choi, 2010). Neurofibrillary tangles in AD brains are aggregates of the tau protein, which is a microtubule-binding protein that has been hyper-phosphorylated by several kinases including c-jun N-terminal kinase (JNK), p38 MAPK and extracellular signal-regulated kinase1/2 (ERK1/2; Pérez et al., 2008; Wang and Liu, 2008). Dimerization of β-amyloid precursor protein (APP) activates the apoptosis signal-regulating kinase1 (ASK1)-mitogen-activated kinase kinase 6 (MKK6)-p38 cascade, which subsequently phosphorylates tau, resulting in neurofibrillary tangles (Peel et al., 2004).

In neurons, p38MAPK and JNK signaling pathways are activated by oxidative stress, and reactive oxygen species (ROS) is associated with the activation of MAPK pathways in multiple cell types (Ruffels et al., 2004; Son et al., 2011). The activated MAPKs signaling pathways are associated with the pathogenic mechanisms of AD including neuronal apoptosis and transcription of β- and γ-secretases. However, the expression of β-Secretase, whose activity promotes the production of Aβ, is negatively regulated by ERK (Tamagno et al., 2009), and exposure of oligomeric Aβ lead to phosphorylation of ERK1/2 in SH-SY5Y cells (Young et al., 2009).

Cilostazol (CSZ), a selective inhibitor of phosphodiesterase type-3 (PDE3), is an antiplatelet agent and is widely prescribed for improvement of chronic artery obstruction and intermittent claudication. It is used for the prevention of ischemic stroke in Japan and in other Asian countries (Hiatt, 2005; Hankey and Eikelboom, 2010). Clinical research has also shown that treatment with CSZ is effective in preventing recurrent stroke (Shi et al., 2014) and therapy with CSZ improves the cognitive impairments in patients with stable AD receiving acetylcholinesterase inhibitors (Tai et al., 2017). Interestingly, in a mice model of cerebral amyloid angiopathy, CSZ significantly improves the clearance of fluorescent soluble Aβ tracers (Maki et al., 2014). In humans the mRNA expression of PDE3A and PDE3B in the brain is substantially low whereas the mRNA expression of PDE3A is highest in the heart (Lakics et al., 2010). Therefore, it is unlikely that the inhibitory effect of CSZ on PDE3 would be a major contributing mechanism to the improvement of cognitive impairments. That is, the mechanism by which CSZ imparts cell protection is most likely independent of PDE3 action.

The objective of this study is to elucidate the molecular mechanisms behind the protective effect of CSZ on Aβ-induced neurotoxicity triggered by oxidative stress, and to investigate whether the MAPKs signaling pathway contributes to neuroprotection.

## Materials and Methods

### Drugs and Reagents

Amyloid β-protein (Aβ, Human, 1–42) was purchased from Peptide Institute, Incorporation (Osaka, Japan). Monomeric amyloid β1–42 (Aβ) was incubated for 24 h at 37°C to allow self-aggregation and oligomerization before treatment. Cilostazol (CSZ), all-trans-retinoic acid (ATRA) and Dulbecco’s modified Eagle’s medium (DMEM Ham’s F-12) were purchased from Wako Company (Osaka, Japan). Fetal bovine serum (FBS), an inhibitor of MAPK/ERK kinase1/2 (MEK1/2; PD98059), and an inhibitor of p38 MAPK (SB202190) were purchased from Sigma-Aldrich Company (MO, USA). Penicillin G sodium, streptomycin sulfate and amphotericin B were obtained from Life Technologies (Camarillo, CA, USA). All other chemicals used in this experiment were of the purest grade commercially available.

### Cell Culture and Drug Treatment

SH-SY5Y cells (human neuroblastoma, EC-94030304) were obtained from “The European Collection of Authenticated Cell Cultures” (ECACC, London, UK). SH-SY5Y cells are comparable to neurons in their morphological and neurochemical properties, and have been widely used as a model in studies involving neuronal injury or death, including neurodegenerative diseases, cerebral ischemia/reperfusion, and epilepsy (Liang et al., 2013; Han et al., 2015). SH-SY5Y cells were cultured in DMEM Ham’s F-12 containing 10% FBS and penicillin G sodium, streptomycin sulfate, and amphotericin B, and maintained in a humidified atmosphere of 5% CO_2_ and 95% air at 37°C. In this study, SH-SY5Y cells were first differentiated with ATRA, and subsequently the differentiated cells were cultured with or without Aβ (used as a control) for 20 h at 37°C. The reason why we chose 20 h as the most preferable incubation time for our study is based on our preliminary study. In AD, neurotoxicity of Aβ results from a long-time accumulation of low-level Aβ, not from a short-time accumulation of high-level Aβ. In preliminary experiments, only weak toxicity was observed as the viability was 86.1 ± 0.4% (mean ± SEM) when SH-SY5Y cells was treated with 2.5 μM Aβ for a short time (3 h). The optimal condition of 40%–50% cytotoxicity was to treat the cells with 2.5 μM Aβ for 20 h. All treatments were carried out under sterile conditions.

### Cell Viability Assay

**Figure 1:**
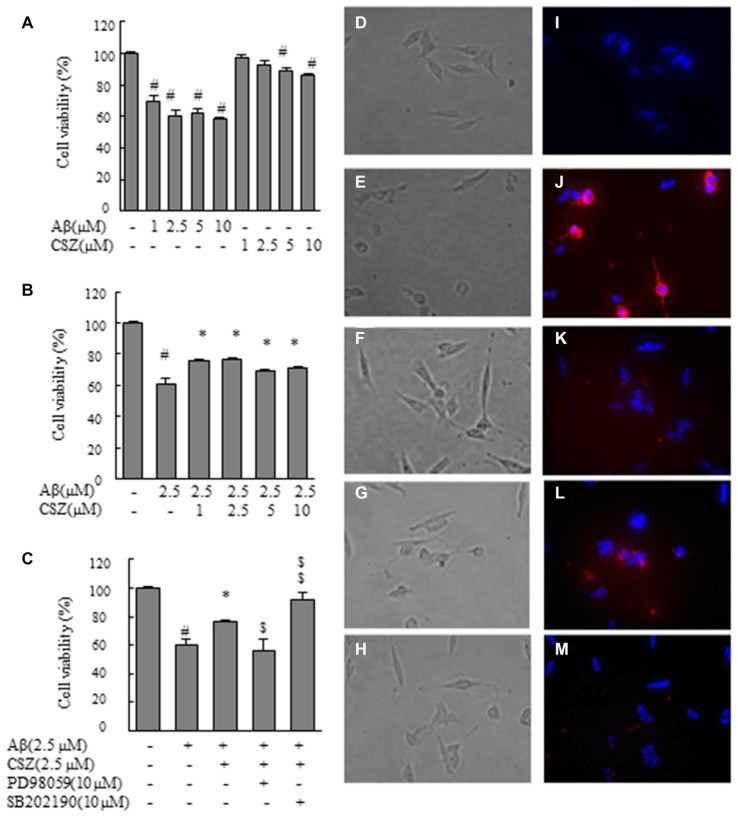
Effect of cilostazol (CSZ) on viability and staining with annexin V and Hoechst33342 in amyloid β (Aβ)-stimulated SH-SY5Y cells. We evaluated the effect of Aβ on cell viability using MTT assay and stained with annexin V and Hoechst33342. **(A)** Cell viability of SH-SY5Y cells treated with Aβ and CSZ for 20 h. **(B)** The effect of Aβ with or without CSZ on cell viability of SH-SY5Y cells. **(C)** The effect of Aβ and CSZ with or without the inhibitor of MAPK/ERK kinase1/2 (MEK1/2) (PD98059) or p38 mitogen-activated protein kinases (MAPK) (SB202190) on cell viability of SH-SY5Y cells. Cells were observed under a phase-contrast microscope **(D–H)** and a fluorescence microscope **(I–M)**. Images **(D,I)** untreated SH-SY5Y cells; images **(E,J)** SH-SY5Y cells treated with Aβ; and images **(F,K)**: SH-SY5Y cells treated with Aβ + CSZ; images **(G,L)** SH-SY5Y cells treated with Aβ + CSZ + inhibitor of MEK1/2; images **(H,M)** SH-SY5Y cells treated with Aβ + CSZ + inhibitor of p38 MAPK. Values are expressed as mean + SEM. ^#^Compared vs. respective controls (*p* < 0.01). *Compared vs. Aβ-treated cells (*p* < 0.05). ^$^Compared vs. Aβ + CSZ-treated cells (*p* < 0.05). ^$$^Compared vs. Aβ + CSZ-treated cells (*p* < 0.01).

We measured cell viability on the basis of blue formazan formation metabolized from colorless MTT (3-(4,5-dimethylthiazol-2-yl)-2,5-diphenyltetrazolium bromide) by mitochondrial dehydrogenases, which are active only in live cells. In order to arrive at a suitable concentration of Aβ to induce cytotoxicity in SH-SY5Y cells, we conducted a preliminary experiment in which SH-SY5Y cells were treated with Aβ (1, 2.5, 5, or 10 μM), and found that 2.5 μM of Aβ was optimal (Figure 1A). Likewise, to study the protective effects of CSZ against Aβ-induced cytotoxicity, we pretreated SH-SY5Y cells with CSZ (1, 2.5, 5, or 10 μM) for 1 h followed by treatment with Aβ (2.5 μM) + CSZ (the concentration used for pretreatment) for 20 h. After incubation, the supernatant was removed and formation of formazan, using Cell Proliferation Kit I (Roche Diagnostics GmbH, Mannheim, Germany), was measured at 540 nm using a microplate reader Spectra Max i3 (Molecular Devices Co., Sunnyvale, CA, USA). We found that 2.5 μM of CSZ is optimal in reversing the Aβ-induced cytotoxicity. Therefore, throughout this study, we used Aβ and CSZ at 2.5 μM each.

### Staining with Annexin V and Hoechst33342

SH-SY5Y cells cultured in 6-well plates and treated with Aβ (2.5 μM) and CSZ (2.5 μM) for 20 h were stained with a DNA dye, Hoechst33342 (Wako, Osaka, Japan) to visualize nuclear morphology. Stained cells were then washed with phosphate-buffered saline (PBS), and specific binding of annexin V-cy3 (Annexin V-cy3 Apoptosis Detection Kit; Medical and Biological Laboratories, Nagoya, Japan) was carried out by incubating the cells for 5 min at room temperature in binding buffer containing annexin V. This kit detects the distribution of phosphatidylserine in the outer monolayer of the cell membrane, and found in the early stage of apoptosis, using fluorescence emitted from specific Cy3-labeled annexin V. After 20 h of incubation with Aβ, SH-SY5Y cells were stained according to the manufacturer’s manual, and examined under a fluorescence microscope (DIAPHOT TMD 300, Nikon Co. Ltd., Tokyo, Japan) for stained cells in the early stages of apoptosis.

### Detection of Caspase-3 and -9 Activities

Activities of caspase-3 and caspase-9 were determined fluorometrically using the respective synthetic peptide substrates obtained from Kamiya Biomedical Company (WA, USA). SH-SY5Y cells were incubated, with or without pretreatment with CSZ (2.5 μM), for 1 h followed by treatment with Aβ + CSZ for 20 h. After incubation, the cells were rinsed with cold PBS and resuspended in chilled cell lysis buffer (Cell Signaling Technology, Inc., MA, USA), incubated for 10 min on ice, and then centrifuged at 10,000× *g* for 3 min. The supernatants were then added to the reaction buffer containing 10 μM dithiothreitol (DTT; Medical and Biological Laboratories Co. Ltd., Aichi, Japan) and the respective specific peptide substrate and incubated at 37°C. Substrates (Kamiya Biochemical Company, Seattle, WA, USA) used for caspase-3 and caspase-9 were Asp-Glu-Val-Asp-7-amino-4-trifluoromethyl coumarin (DEVD-AFC) and Leu-Glu-His-Asp-AFC (LEHD-AFC), respectively. AFC released by enzyme reaction was measured spectrophotometrically (excitation wavelength: 405 nm; emission wavelength: 505 nm) using the Spectra Max i3 (Molecular Devices Co., Sunnyvale, CA, USA).

### Detection of Reactive Oxygen Species (ROS)

To study the effect of Aβ treatment on hydrogen peroxide production, we used CM-H_2_DCFDA, a useful indicator for ROS detection. SH-SY5Y cells were seeded in 96-well plates at 1 × 10^5^ cells/ml and incubated as described in “Cell Culture and Drug Treatment” section. We used the Spectra Max i3 (Molecular Devices Co., Sunnyvale, CA, USA) to determine the fluorescence intensity at excitation and emission wavelengths of 488 and 525 nm, respectively.

### Assay of Nicotinamide Adenine Dinucleotide Phosphate Oxidase (Nox) Activity

NOX activity was measured by using the lucigenin-enhanced chemiluminescence method as described elsewhere (Block et al., 2007). Lucigenin is a luminescence-generating reagent that interacts with free radicals, such as superoxide anion, and the emitted luminescence can be quantitatively measured using a luminometer. SH-SY5Y cells were washed with ice-cold PBS and disrupted by sonication in cell lysis buffer. The cell lysate was resuspended with luminescence buffer (Krebs buffer containing 0.5 mM lucigenin), before adding nicotinamide adenine dinucleotide phosphate (NADPH; 0.1 mM) as the substrate. The emitted luminescence was detected by a luminometer. Briefly, photon emission from lucigenin as an acceptor of electron/O_2_− generated by the NADPH oxidase complex was measured every 30 s for 30 min in the Spectra Max i3 (Molecular Devices Co., Sunnyvale, CA, USA). The data were converted to relative light units (RLU)/min/mg of protein. Protein content was determined using the Bio-Rad protein assay reagent.

### Semiquantitative Reverse-Transcription Polymerase Chain Reaction (RT-PCR) Analysis

To measure mRNA expressions of NOX-4 and β-actin, SH-SY5Y cells were pretreated with CSZ (2.5 μM) for 1 h and then treated with Aβ (2.5 μM) plus CSZ (2.5 μM) for another 20 h. After treatment, total RNA was extracted from SH-SY5Y cells using the RNeasy RNA Mini kit (Qiagen K.K., Tokyo, Japan). The reverse-transcription polymerase chain reaction (RT-PCR) analysis was carried out using the Omniscript RT Kit (Qiagen K.K.) according to the manufacturer’s instructions. The sequence of the primers used for amplification were: NOX4-F: 5′-CCACAGACTTGGCTTTGGAT; NOX4-R: 5′-GGCAGAATTTCGGAGTCTTG, and β-actin-F: 5′-TCGTCACCAACTGGGACGACATGG; β-actin-R: 5′-GATCTTGATCTTCATTGTGCTGGG. PCR Amplification for NOX-4 included 38 amplification cycles (one cycle comprised of 94°C for 30 s, 55°C for 30 s and 72°C for 30 s), followed by a final extension at 72°C for 7 min. The reaction (30 cycles) for β-actin comprised treatment at 95°C for 1 min before 60°C for 1 min followed by 72°C for 2 min for each cycle. Each sample was tested in triplicate. The number of cycles for each product was determined on the basis of kinetic studies. Some samples were picked up after every two cycles, and the PCR products were electrophoresed on 2% agarose gels and visualized by ultraviolet transillumination (Atto Corp, Tokyo, Japan). Bands were quantified by densitometry using a scanner employing Scion Image Version 4.02 software (NIH, USA). Reaction cycle-PCR product yield curves of the respective reaction mixture were plotted on graphs. The linear portion of the fluorescence intensity curve was used to extrapolate the data. The ratio of target cDNA to β-actin was used as a relative estimate of mRNA abundance.

### Assay of MAPK Phosphorylation

We studied the role of MAPK in Aβ-induced neurotoxicity in SH-SY5Y cells by examining their phosphorylation activities. For examination of the phosphorylation activities of ERK1/2 and p38 MAPK, we used Cell-based ERK1/2 (Thr202/Tyr204) ELISA kit and Cell-based p38 MAPK (Thr180/Tyr182) ELISA kit. SH-SY5Y cells were incubated with or without pretreatment with CSZ (2.5 μM) for 1 h followed by treatment with Aβ (2.5 μM) + CSZ (2.5 μM) for 30 min. Cells were fixed and blocked as directed in the manual, and then incubated with anti-phospho-ERK1/2 (Thr202/tyr204) or anti-ERK1/2 and anti-phospho-p38 MAPK (Thr180/Tyr182) or anti-p38 MAPK, respectively. The cells were washed, and Horseradish Peroxidase (HRP)-conjugated anti-mouse IgG (secondary antibody) was added. The cells were washed again, and TMB (3,3′,5,5′-tetramethylbenzidine) substrate solution was added, resulting in the development of color in proportion to the amount of protein. The reaction was stopped by adding the stop solution of the assay kit, and the intensity of the color was measured at 450 nm.

### Detection of Phosphorylation of Cyclic Adenosine Monophosphate Response Element Binding Protein (CREB)

Cyclic adenosine monophosphate response element-binding protein (CREB) which is activated by phosphorylation at residue Ser-133 is one of the best characterized transcription factor, an event induced by various extracellular signals (Lonze and Ginty, 2002). SH-SY5Y cells were lysed in a cytoplasmic extraction buffer and centrifuged. Protein concentrations of the samples were then determined using the DC Protein Assay (Bio-Rad Laboratories). To examine the phosphorylation activities of CREB, we used CREB (Total) Instant OneTM ELISA kit and CREB (Phospho) Instant OneTM ELISA kit (Affymetrix, CA, USA).

### Assay of Bax, Bcl-2 and Cytochrome c

After treatment with Aβ (2.5 μM) for 20 h with or without 1 h pretreatment with CSZ (2.5 μM) or one of inhibitors of p38 MAPK or MEK1/2, cells were extracted with a cell lysis buffer (100 mM PBS, 150 mM NaCl, 1 mM ethylenediaminetetraacetic acid (EDTA), 0.5% TritonX-100, 0.005% Tween 20, 1 mM Phenylmethylsulfonyl fluoride, and Protease Inhibitor (diluted 100-fold)). For the quantitative determination of Bax and Bcl-2 in SH-SY5Y cells lysates, Bax human EIA kit (Enzo Life Sciences Int’l, Inc., NY, USA) and Human Bcl-2 Platinum ELISA kit (eBioscience, Vienna, Austria) were used, respectively.

The cytosolic fraction of SH-SY5Y cells was extracted with mitochondria/cytosol fraction kit (BioVision Research Inc., Milpitas, CA, USA). Cytochrome c levels in cytosolic fractions was evaluated using a detection kit (Quantikine ELISA, human cytochrome c, R&D systems Inc., MN, USA).

### Assay of Cu/Zn- Superoxide Dismutase (SOD) and SOD Activity

Cu/Zn-Superoxide Dismutase (SOD) isozyme contents in the cell lysate were determined by ELISA using monoclonal antibody (Cu, Zn-SOD ELISA System; Amersham Pharmacia Biotec Co., Tokyo, Japan). These ELISA systems showed no cross-reactivity with other SOD isozymes. SOD activity was determined as the inhibition of chromagen reduction by using OxiSelectTM SOD Activity Assay (Cell Biolabs, Inc., San Diego, CA, USA).

### Statistical Analysis

Each measurement was repeated in triplicates. Results are expressed as the mean + SEM We used one-way ANOVA, to compare the effects of various treatments with the untreated control cells, followed by Dunnett’s *post hoc* test. Different *p-value* of less than 0.05 was considered statistically significant.

## Results

### Effect of CSZ on Viability and Staining with Annexin V and Hoechst33342 in Aβ-stimulated SH-SY5Y Cells

First, we examined the effect of Aβ on cell viability (Figures 1A–C). MTT assay revealed that Aβ (1, 2.5, 5 and 10 μM) exposure for 20 h decreased cell viability in a dose-dependent manner. Treated cells with 1 or 2.5 μM of CSZ had no significant effect compared with the control cells. However, 5 or 10 μM of CSZ significantly reduced the cell viability (Figure 1A). Based on these results, we selected 2.5 μM of Aβ and 2.5 μM of CSZ for our following experiment. Pretreatment with CSZ for 1 h significantly prevented cell death that was induced by treatment with Aβ alone (Figure 1B). Furthermore, the cell viability was significantly suppressed when SH-SY5Y cells were pretreated with an inhibitor of MEK1/2 (PD98059) compared to cells treated with Aβ + CSZ, while pretreatment with an inhibitor of p38 MAPK (SB202190) significant increased it (Figure 1C).

Figures 1D–H shows the cells observed under a phase-contrast microscope. Figure 1D shows the control cells. Treatment of SH-SY5Y cells with Aβ (2.5 μM) induced extensive damages in dendrite morphology as seen in their shortened or branchless forms (Figure 1E). However, pretreatment with CSZ suppressed the dendrite damage after Aβ (2.5 μM) treatment (Figure 1F). A well-known feature of an early apoptotic process is the translocation of phosphatidylserine from the inner leaflet to the outer leaflet on the membrane bilayer. The results obtained with the annexin V-Cy3 binding assay of untreated and treated cells are presented in Figures 1I–M. Red fluorescence emission and nuclear condensation show that apoptosis was induced in Aβ-treated cells (Figure 1J). However, the both red and bright blue fluorescence were attenuated in the CSZ-pretreated cells compared with those in the Aβ-treated cells (Figure 1K). Furthermore, pretreatment with an inhibitor of MEK1/2 (PD98059) considerably increased the number of annexin V-positive cells compared to cells that were treated with Aβ + CSZ (Figure 1L), but pretreatment with an inhibitor of p38 MAPK (SB202190) did not (Figure 1M).

### Effect of CSZ on Caspase-3 and -9 Activities in Aβ-stimulated SH-SY5Y Cells

**Figure 2:**
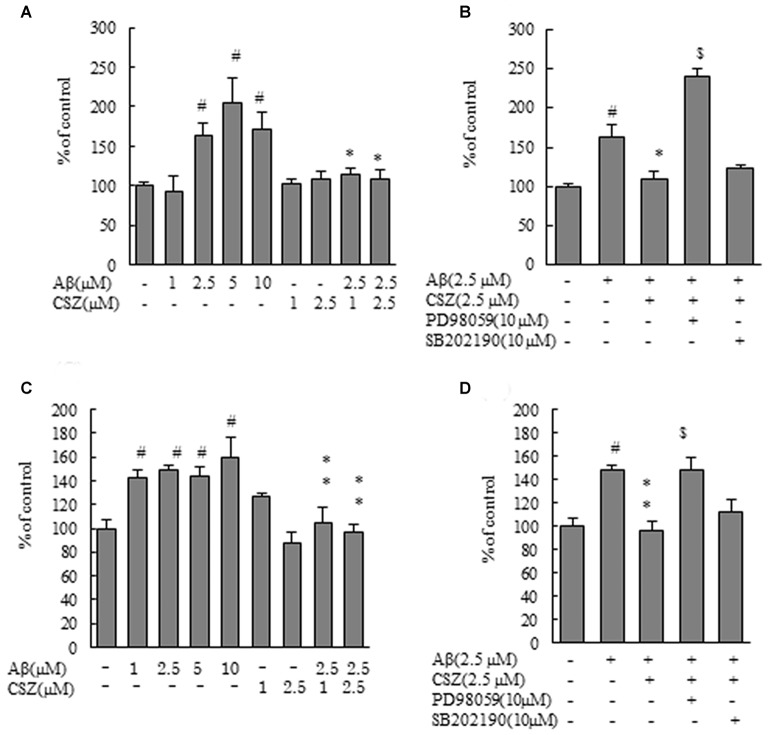
Effects of CSZ on caspase-3 and -9 activitives in Aβ-stimulated SH-SY5Y cells. Caspase-3 and -9 activities were measured using each peptide substrate (DEVD-AFC and LEHD-AFC). **(A)** Caspase-3 activity in SH-SY5Y cells treated with Aβ (1–10 μM) or CSZ (1, 2.5 μM) for 20 h. **(B)** Caspase-3 activity was determined in SH-SY5Y cells incubated with or without 1 h pretreatment with CSZ or inhibitor of MEK1/2 or p38 MAPK followed by 20 h incubation with Aβ. Caspase-3 activity of the control cells was 60.54 ± 4.35 nmol/mg protein/h. **(C)** Caspase-9 activity in SH-SY5Y cells treated with Aβ (1–10 μM) or CSZ (1, 2.5 μM) for 20 h. **(D)** Caspase-9 activity was determined in SH-SY5Y cells incubated with or without 1 h pretreatment with CSZ or inhibitor of MEK1/2 or p38 MAPK followed by 20 h incubation with Aβ. Caspase-9 activity of the control cells was 25.72 ± 3.2 nmol/mg protein/h. Values are expressed as mean + SEM. ^#^Compared vs. respective controls (*p* < 0.01). *Compared vs. Aβ-treated cells (*p* < 0.05). **Compared vs. Aβ-treated cells (*p* < 0.01). ^$^Compared vs. Aβ + CSZ-treated cells (*p* < 0.01).

Caspases play a critical role in the apoptosis of SH-SY5Y cells. therefore, we next measured caspase-3 and -9 activities using synthetic fluorometric substrates (Figure 2). After incubation with Aβ (2.5, 5 and 10 μM) for 20 h, the activities of caspase-3 and caspase-9 significantly increased compared with the controls. Pretreatment with CSZ (1 and 2.5 μM) significantly prevented the increase in caspase-3 and caspase-9 activities compared to cells treated with Aβ alone (Figures 2A,C). As shown in Figures 2B,D, both caspase activities significantly increased with an inhibitor of MEK1/2 (PD98059) pretreatment, indicating that mitochondria are involved in the apoptosis induced by the Aβ treatment and that ERK is acting in the upstream of mitochondrial involvement.

### Effect of CSZ on ROS Production in Aβ-stimulated SH-SY5Y Cells

**Figure 3:**
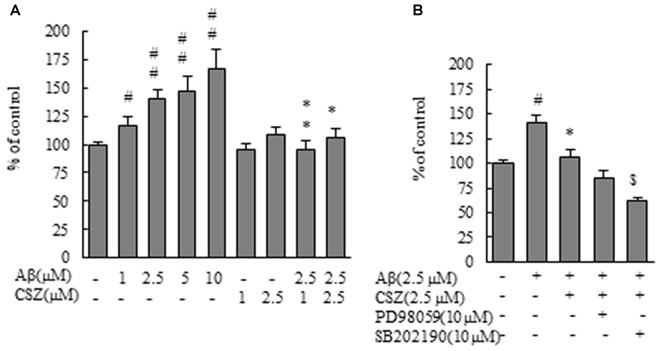
Effect of CSZ on reactive oxygen species (ROS) generation in Aβ-stimulated SH-SY5Y cells. ROS generation in SH-SY5Y cells was evaluated using CM-H2DCFDA. **(A)** The generation of ROS in SH-SY5Y cells treated with Aβ (1–10 μM) or CSZ (1, 2.5 μM) for 1 h. **(B)** The generation of ROS was determined in SH-SY5Y cells incubated with or without 1 h pretreatment with CSZ or 10 μM inhibitor of MEK1/2 or p38 MAPK followed by 1 h incubation with Aβ. ROS generation of the control cells was 1455290 ± 70201 Fluorescence intensity. Values are expressed as mean + SEM. ^#^Compared vs. respective controls (*p* < 0.05). ^##^Compared vs. respective controls (*p* < 0.01). *Compared vs. Aβ-treated cells (*p* < 0.05). **Compared vs. Aβ-treated cells (*p* < 0.01). ^$^Compared vs. Aβ + CSZ-treated cells (*p* < 0.01).

For investigation of the effect of Aβ treatment on hydrogen peroxide production, we used CM-H2DCFDA, a ROS-sensitive dye. DCF fluorescence as an indicator of ROS formation is shown in Figure 3. ROS production in SH-SY5Y cells treated with Aβ (1–10 μM) increased dose dependently compared with control cells. However, the increase in DCF fluorescence upon exposure to Aβ was significantly eliminated by pretreatment with CSZ (1 and 2.5 μM; Figure 3A). Furthermore, the ROS production was significantly suppressed when SH-SY5Y cells were pretreated with an inhibitor of p38 MAPK (SB202190) compared to cells treated with Aβ + CSZ (Figure 3B). The effect of an MEK1/2 inhibitor and the related actions of ERK are rather paradoxical in neuronal cells, and we will mention the issue in the “Discussion” section.

### Effect of CSZ on Nox Activity and Nox4 mRNA Expression in Aβ-stimulated SH-SY5Y Cells

**Figure 4:**
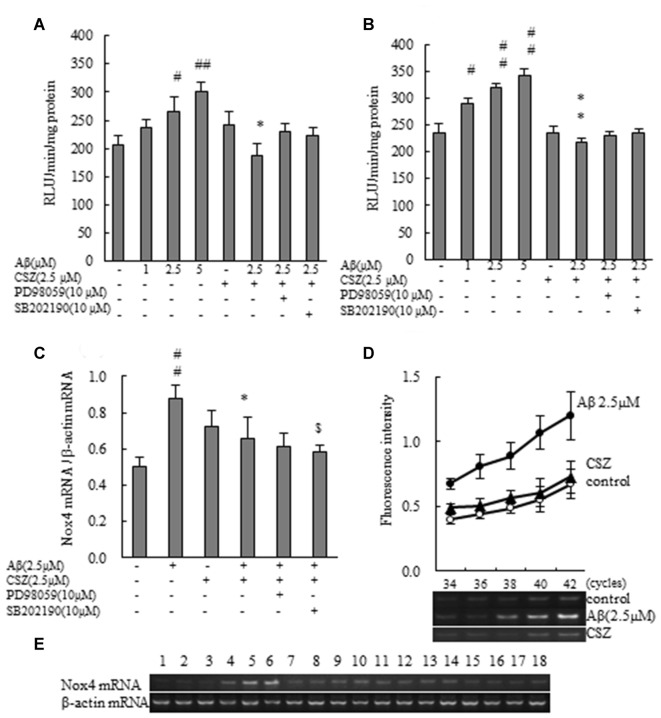
Effect of CSZ on Nox activity and the mRNA expression analysis of NOX4 in Aβ-stimulated SH-SY5Y cells. **(A)** Nox activities were determined in SH-SY5Y cells incubated with or without 1 h pretreatment with CSZ or inhibitor of MEK1/2 or p38 MAPK followed by 1 h incubation with Aβ. Values are expressed as mean + SEM. **(B)** Nox activities were determined in SH-SY5Y cells incubated with or without 1 h pretreatment with CSZ or inhibitor of MEK1/2 or p38 MAPK followed by 20 h incubation with Aβ. Values are expressed as mean + SEM. **(C)** The mRNA expression analysis of NOX4 in treated SH-SY5Y cells are examined. The effects of respective inhibitor were also examined. Values are expressed as mean + SEM. **(D)** Reaction cycles-PCR product yield curves of each reaction mixture were plotted. **(E)** Picture shows the bands obtained in agarose gel electrophoresis for Nox4 and β-actin. Lane1–3: control, Lane4–6: 2.5 μM Aβ, Lane7–9: 2.5 μM CSZ, Lane10–12: 2.5 μM Aβ + 2.5 μM CSZ, Lane13–15: 2.5 μM Aβ + 2.5 μM CSZ + 10 μM PD98059, Lane16–18: 2.5 μM Aβ + 2.5 μM CSZ + 10 μM SB202190. ^#^Compared vs. respective control cells (*p* < 0.05). ^##^Compared vs. respective control cells (*p* < 0.01). *Compared vs. Aβ-treated cells (*p* < 0.05). **Compared vs. Aβ-treated cells (*p* < 0.01). ^$^Compared vs. Aβ + CSZ-treated cells (*p* < 0.05).

We evaluated the effect of CSZ on Nox activity in Aβ-stimulated SH-SY5Y cells. First, we measured the effect of Aβ on Nox activation. Exposure of SH-SY5Y cells to Aβ resulted in a dose-dependent increase in Nox activity. As shown in Figures 4A,B, Aβ exposure induced a robust increase in Nox activities for 1 and 20 h, respectively, which was suppressed by pretreatment with CSZ for 1 h and 20 h of incubation, respectively. However, neither of the MAPK inhibitors altered the CSZ effect on Aβ-induced NOX activity.

The mRNA expression analysis of NOX4 in treated SH-SY5Y cells is shown in Figures 4C–E. The amount of target mRNA expression increased with cycle numbers of PCR (Figure 4D), and PCR products were semi-quantified during the reaction by measuring the fluorescence intensity. The NOX4 mRNA expression levels in Aβ-treated SH-SY5Y cells increased significantly compared to the control cells (Figures 4C,E). In contrast, pretreatment with CSZ prevented these Aβ-induced increases in NOX4 mRNA expression levels. Furthermore, pretreatment with p38 MAPK inhibitor suppressed the CSZ effect on Aβ-induced Nox4 mRNA expression level.

### Effects of CSZ on Phosphorylation of ERK1/2, p38 MAPK and CREB in Aβ-stimulated SH-SY5Y Cells

**Figure 5:**
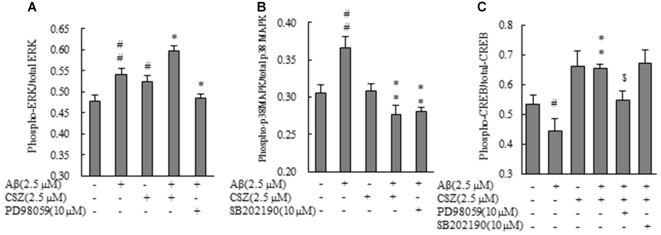
Effects of CSZ on phosphorylation of extracellular signal-regulated kinase1/2 (ERK1/2), p38 MAPK and cyclic adenosine monophosphate response element-binding protein (CREB) in Aβ-stimulated SH-SY5Y cells. Phosphorylation of ERK1/2, p38 MAPK and CREB were examined using ELISA kit described in “Materials and Methods” section. **(A)** ERK1/2 phosphorylation. **(B)** p38 MAPK phosphorylation. SH-SY5Y cells were incubated with or without pretreatment with CSZ for 1 h followed by treatment with Aβ for 30 min. The effects of respective inhibitor were also examined. **(C)** CREB phosphorylation. Values are expressed as mean + SEM ^#^Compared vs. respective control cells (*p* < 0.05). ^##^Compared vs. respective control cells (*p* < 0.01). *Compared vs. Aβ-treated cells (*p* < 0.05). **Compared vs. Aβ-treated cells (*p* < 0.01). ^$^Compared vs. Aβ + CSZ-treated cells (*p* < 0.01).

Phosphorylation activities of ERK1/2 and p38 MAPK were compared in cells treated with or without Aβ in the presence or absence of CSZ pretreatment. Phosphorylated ERK1/2 increased in Aβ-treated SH-SY5Y cells compared to untreated cells, furthermore, pretreatment with CSZ showed an extensive increase in ERK phosphorylation. Pretreatment with MEK1/2-inhibitor was significantly decreased the Aβ-induced ERK phosphorylation (Figure 5A). As shown in Figure 5B, phosphorylation of p38 MAPK showed a significant increase in Aβ-treated cells, while pretreatment with CSZ significantly suppressed this effect.

To study the effects of CSZ on the phosphorylation of CREB in Aβ-induced neurotoxicity, we first examined how CSZ affected total CREB and phosphorylated CREB protein levels. As shown in Figure 5C, exposure of Aβ to SH-SY5Y cells for 20 h caused a reduction in the ratio of phosphorylated CREB/total CREB, and pretreatment with CSZ prevented this.

### Effects of CSZ on Bax, Bcl-2 and Cytochrome c in Aβ-stimulated SH-SY5Y Cells

**Figure 6:**
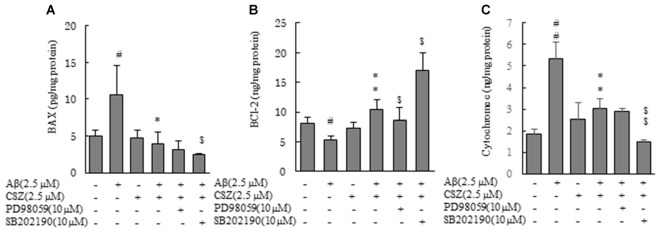
Effects of CSZ and inhibitors of MEK1/2 and p38 MAPK on Bax, Bcl-2 and cytochrome c in Aβ- timulated SH-SY5Y cells. Levels of Bax and Bcl-2 were determined in SH-SY5Y cells incubated with or without 1 h pretreatment with CSZ or inhibitor of MEK1/2 or p38 MAPK followed by 20 h incubation with Aβ. Cells were extracted with a cell lysis buffer and quantitative determination of Bax, Bcl-2 and cytochrome c. **(A)** Bax **(B)** Bcl-2 **(C)** cytochrome c. Values are expressed as mean + SEM ^#^Compared vs. control cells (*p* < 0.05). ^##^Compared vs. control cells (*p* < 0.01). *Compared vs. Aβ-treated cells (*p* < 0.05). **Compared vs. Aβ-treated cells (*p* < 0.01). ^$^Compared vs. Aβ + CSZ-treated cells (*p* < 0.05). ^$$^Compared vs. Aβ + CSZ-treated cells (*p* < 0.01).

Earlier we demonstrated that the mitochondria was associated with an Aβ-induced apoptotic pathway because caspase-9 activity significantly elevated in the Aβ-treated cells (Figure 2). We examined levels of two proteins of Bcl-2 family, Bax (proapoptotic protein) and Bcl-2 (antiapoptotic protein), in regulating release of the cytochrome c from mitochondria. SH-SY5Y cells were treated with or without Aβ in the presence and absence of pretreatment with CSZ or inhibitors of MAPK (PD98059 and SB202190; Figure 6). The levels of Bax and cytochrome c in the Aβ-treated cells significantly increased, which was prevented by pretreatment with CSZ. Pretreatment with the p38MAPK inhibitor showed a significant decrease in Bax and cytochrome c levels compared to treatment with Aβ + CSZ (Figures 6A,C). On the other hand, the level of Bcl-2 in the Aβ-treated cells was significantly decreased, while the reduction was significantly suppressed by pretreatment with CSZ. The pretreatment with the MEK1/2 inhibitor showed significant decrease in Bcl-2 level compared to treatment of Aβ + CSZ (Figure 6B).

### Effect of CSZ on Cu/Zn-SOD Content and SOD Activity in Aβ-stimulated SH-SY5Y Cells

**Figure 7:**
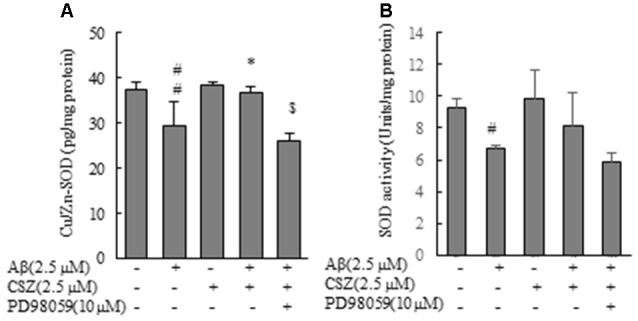
Effects of CSZ on Cu/Zn-Superoxide Dismutase (SOD) content, SOD activity in Aβ-stimulated SH-SY5Y cells. **(A)** Cu/Zn-SOD isozyme contents, **(B)** SOD activities in the cell lysate were determined. The effect of a MEK1/2 or p38 MAPK inhibitor was also examined. Values are expressed as mean + SEM. ^#^Statistical significance compared vs. control cells (*p* < 0.05). ^##^Statistical significance compared vs. control cells (*p* < 0.01). *Statistical significance compared vs. 2.5 μM Aβ-treated cells (*p* < 0.01). ^$^Statistical significance compared vs. 2.5 μM Aβ + CSZ-treated cells (*p* < 0.01).

As shown in Figure 7, Cu/Zn-SOD content and SOD activity in Aβ-induced neurotoxic cells were significantly reduced compared with those of the control cells, and this reduction of Cu/Zn-SOD content was significantly recovered when Aβ-exposed cells were pretreated with CSZ. On the other hand, Cu/Zn-SOD content in Aβ-induced neurotoxic cells after the pretreatment with the inhibitor of MEK1/2 remained unchanged (Figure 7A).

## Discussion

The present study showed that pretreatment with CSZ significantly suppresses Aβ-induced cytotoxicity in SH-SY5Y cells by reducing ROS production, and also by providing protection against mitochondrial dysfunction. The underlying mechanisms behind the protective effects of CSZ on Aβ-induced cytotoxicity in SH-SY5Y cells may be inhibition of oxidative stress and suppression of MAPK activation.

Oxidative stress is considered important according to the pathological hypothesis of neurodegenerative diseases such as AD, Parkinson’s disease and amyotrophic lateral sclerosis. It is found in the early stages of mild cognitive impairment (MCI) in AD progression, along with increased protein levels of oxidative stress markers (Barone et al., 2011, 2012). In an early AD brain, 8-hydroxyguanosine levels are elevated in the hippocampus, frontal, and occipital neocortex, which correlates with the Aβ load (Nunomura et al., 2001). Similar to the report mentioned above, the present study indicated that exposure to Aβ results in oxidative stress to induce an increment in ROS generation, Nox activation, expression of Nox4 mRNA, and reduction of Cu/Zn-SOD, which is the superoxide scavenger enzyme in SH-SY5Y cells.

ROS produced by Noxs, which are membrane-bound multicomponent enzyme complexes, transmit a signal to various molecules. The role of Nox-dependent oxidative stress in the pathogenesis of some neurodegenerative disease was found. Association of Nox isoforms and AD and other neuropathology has extensively studied in both man and animals (Hankey and Eikelboom, 2010; Bruce-Keller et al., 2011).

The present study indicated that exposure to Aβ results in oxidative stress to induce an increment in ROS generation and Nox activation (Figures 3, 4). Oxidative stress caused by Aβ exposure may be associated with Nox enzyme activation.

Furthermore, Aβ exposure increased the release of cytochrome c and Bax, and decreased Bcl-2. The Aβ-induced neurotoxicity in the brains of AD patients is thought to be considerably related to neuronal mitochondrial dysfunction and oxidative stress. Unbalanced population of pro-apoptotic/anti-apoptotic proteins and increased levels of caspases are reported in AD patients’ brain (Pugazhenthi et al., 2011). Therefore, it may be beneficial for patients to quench excess ROS or suppress their generation using antioxidants, because this would prevent neuronal cell death caused by oxidative stress.

CSZ has been reported to be an antioxidant. In the present study, we further studied the role of CSZ in protecting against Aβ-induced oxidative stress and its mechanisms. Generally, patients take 100 mg to CSZ orally twice a day as an antiplatelet drug. Plasma concentration at steady state after multiple dosing of CSZ 100 mg twice daily is between 1.5–3.2 μM. Each tissue concentration of CSZ previously reported using radioactive carbon in the plasma, cerebrum, and hypophysis was 993 ng/ml (2.69 μM), 99 ng/g and 946 ng/g, respectively after an oral administration of C^14^-cilostazol (10 mg/kg) in male rats (Akiyama et al., 1985). The concentrations of CSZ (2.5 μM) that we used in this study are similar to the serum concentration found in clinical settings, and therefore we speculate that this concentration may show the neuroprotective effects in clinical use.

CSZ is a quinolone derivative that is considered as an important structural unit in many antioxidant agents (Orhan Püsküllü et al., 2013). This study shows that treatment with CSZ significantly decreased the activation of Nox and expression of Nox-4 mRNA in the Aβ-exposed cell (Figure 4). Moreover, pretreatment with CSZ resulted in significant reduction of ROS and Bax and in the significant increase in Bcl-2 and Cu/Zn-SOD.

Recent research reported that repeated treatment with CSZ completely prevented the accumulation of malondialdehyde induced by Aβ25–35 and attenuated cognitive impairments (Hiramatsu et al., 2010). Moreover, CSZ reduced infarct volumes in mice with permanent focal ischemia and NOX2-positive neurons (Shichinohe et al., 2015) and inhibited ROS production via inhibition of Nox on murine macrophages (Yun et al., 2009). Similar to these reports, the present results suggested that CSZ attenuated Aβ-induced cytotoxicity in SH-SY5Y cells through inhibition of Nox-derived ROS generation.

In this study, when exposed to Aβ, p38 MAPK was activated and the decreased viability induced by Aβ was largely prevented by CSZ, and this prevention was enhanced by the p38MAPK inhibitor (Figure 1C). Furthermore, pretreatment with the inhibitor of p38 MAPK (SB202190) significantly recovered the decrease in cell viability compared with cells treated with 2.5 μM Aβ alone (data not shown). We found that Aβ-induced p38 MAPK activity mediates the Aβ-induced cell death events. The activated MAPK signaling pathways are thought to be involved in the pathogenesis of AD through multiple pathways including induction of neuronal apoptosis (Wang et al., 2014; Kim and Choi, 2015). MAPK signaling pathways have been shown to be activated by receptor-ligand interactions and by a lot of stressors. In AD brain, ROS activates the JNK and p38 MAPK signaling pathways (Zhu et al., 2002; Tabner et al., 2005). It was reported that ROS activated ERKs, JNKs and p38 MAPKs, but in this study, the ROS production seems to induce the activation of JNK, or p38 MAPKs pathways rather than the ERK pathway. Several studies have indicated a role for ROS, which activated the ERK signaling pathway, in animal cells (Mehdi et al., 2007). It had been widely thought that ERK1/2 generally worked toward the cell survival in contrast to JNK and p38 MAPK that are proapoptotic. However, especially in neuronal cells, this concept is no longer useful. Evidence that shows that ERK1/2 plays a crucial role in promoting neuronal cell death, is increasing (Cagnol and Chambard, 2010; Subramaniam and Unsicker, 2010). According to studies cited in these reviews, ERK1/2 actions are determined spatiotemporally; the magnitude and duration of ERK1/2 activity, as well as its sequestration, determine its cellular function. In this study, Aβ activated ERK in SH-SY5Y cells (Figures 5A,B). This activation might not be required for Aβ-induced cell death because MEK1/2 inhibitor (PD98059) completely inhibited ERK activation, and pretreatment with the inhibitor of MEK1/2 did not show considerable alteration compared to cells that treated with Aβ alone.

Pretreatment with a MEK1/2 inhibitor (PD98059) significantly inhibited the CSZ-mediated survival of Aβ-induced cell death (Figures 1C, 2B,D) but induced only a tendency of inhibition of the increment of antiapoptotic Bcl-2 by CSZ (Figure 6B). It may be suggested that ERK1/2 is a part of the important factors in a cellular signaling event mediating the protective effect of CSZ. Furthermore, CSZ-stimulated phosphorylation of CREB was significantly suppressed by the MEK1/2 inhibitor, indicating the involvement of ERK1/2 in the signaling of CREB phosphorylation (Figure 5C). The CREB is a major positive regulatory site in the Bcl-2 promoter (Xiang et al., 2006). Therefore, in this study, the increment of Bcl-2 by CSZ and its inhibition by the MEK1/2 inhibitor seem to describe the consequence of the CREB phosphorylation (Figure 5C).

The level of cAMP in Aβ-induced neurotoxic cells was significantly decreased compared with that in the control cells (112.8 ± 12.9 vs. 170.0 ± 22.3 pmol/mg protein, *p* < 0.05), and the decrease was not significantly recovered when Aβ-exposed cells were pretreated with CSZ (133.1 ± 23.7 pmol/mg protein). CSZ is a selective inhibitor of PDE3 and increases cAMP, however, in this study, pretreatment with CSZ did not increase in cAMP level. It has been indicated that expressions of PDE3A mRNA and PDE3B mRNA in the human brain were substantially low (Lakics et al., 2010). CSZ may have not only selective PDE3 inhibitory action but also various other effects such as activation of AMP-activated protein kinase (AMPK). In preliminary experiments, SH-SY5Y cells treated with CSZ increased AMPK activation (data not shown), AMPK activation by CSZ treatment seems to be independent of cAMP signaling pathway (Suzuki et al., 2008). However, we have not been able to examine whether the NOX suppressive effects of CSZ is independent of other effects of CSZ. From our experiences in apoptosis study of hepatic cells and SH-SY5Y cells, we thought it may be mediated by MAPK such as ERK. The suppressive effect of CSZ on oxidative stress elicited by Aβ demonstrated in our data suggests the possibility that neurotoxicity of Aβ may be suppressed by CSZ. Further detailed investigation is needed to reveal the full mechanism of actions of CSZ.

In conclusion, the present study demonstrates that CSZ inhibits Aβ-induced oxidative stress by suppression of Nox activity in SH-SY5Y cells. CSZ suppressed Aβ-induced P38 MAPK activation and increase in ERK1/2 activity in SH-SY5Y cells. Furthermore, CSZ protects against mitochondrial dysfunction by suppressing Aβ-induced upregulation of Bax and caspase-9 activity, and downregulation of Bcl-2. Thus, CSZ is considered to be a beneficial agent that works by protecting against oxidative stress, mitochondrial dysfunction, and apoptosis, and may serve as a promising therapeutic agent in preventing neuronal damage and as a potential candidate for the treatment of cognitive deficits in AD patients.

## Author Contributions

TO, RO, HS, MS, MI, YM and TY performed all the experiments. TO, RO and MT designed the experiments. TO and MT wrote the article. KO and YK directed the study. All authors read and approved the final manuscript.

## Conflict of Interest Statement

The authors declare that the research was conducted in the absence of any commercial or financial relationships that could be construed as a potential conflict of interest.

## Abbreviations

Aβ: amyloid β
AD: Alzheimer’s disease
AMPK: AMP-activated protein kinase
APP: β-amyloid precursor protein
ASK: apoptosis signal-regulating kinase
ATRA: all-trans-retinoic acid
CREB: cyclic adenosine monophosphate response element-binding protein
CSZ: cilostazol
DTT: dithiothreitol
EDTA: ethylenediaminetetraacetic acid
ERK: extracellular signal-regulated kinase
FBS: Fetal bovine serum
HRP: Horseradish Peroxidase
JNK: c-jun N-terminal kinase
MAPK: mitogen-activated protein kinases
MCI: mild cognitive impairment
MEK: MAPK/ERK kinase
MKK: mitogen activated kinase kinase
MTT: 3-(4,5-dimethylthiazol-2-yl)-2,5-diphenyltetrazolium bromide
NADPH: nicotinamide adenine dinucleotide phosphate
NOX: nicotinamide adenine dinucleotide phosphate oxidase
PBS: phosphate-buffered saline
PCR: polymerase chain reaction
PDE: phosphodiesterase
RLU: relative light units
ROS: reactive oxygen species
SOD: Superoxide dismutase
TMB: 3,3′,5,5′-tetramethylbenzidine.
